# Newborn Screening for Sickle Cell Disease in the Caribbean: An Update of the Present Situation and of the Disease Prevalence

**DOI:** 10.3390/ijns5010005

**Published:** 2019-01-08

**Authors:** Jennifer Knight-Madden, Ketty Lee, Gisèle Elana, Narcisse Elenga, Beatriz Marcheco-Teruel, Ngozi Keshi, Maryse Etienne-Julan, Lesley King, Monika Asnani, Marc Romana, Marie-Dominique Hardy-Dessources

**Affiliations:** 1 Caribbean Institute for Health Research—Sickle Cell Unit, The University of the West Indies, Mona, Kingston 7, Jamaica; 2 Laboratory of Molecular Genetics, Academic Hospital of Guadeloupe, 97159 Pointe-à-Pitre, Guadeloupe; 3 Referral Center for Sickle Cell Disease, Department of Pediatrics, Academic Hospital of Martinique, 97261 Fort de France, Martinique, France; 4 Referral Center for Sickle Cell Disease, Department of Pediatric Medicine and Surgery, Andrée Rosemon General Hospital, 97306 Cayenne, French Guiana, France; 5 National Center of Medical Genetics, 11300 La Habana, Cuba; 6 Paediatric Department, Scarborough General Hospital, 00000 Scarborough, Tobago; 7 Referral Center for Sickle Cell Disease, Sickle Cell Unit, Academic Hospital of Guadeloupe, 97159 Pointe-à-Pitre, Guadeloupe, France; 8 UMR Inserm 1134 Biologie Intégrée du Globule Rouge, Inserm/Université Paris Diderot—Université Sorbonne Paris Cité/INTS/Université des Antilles, Hôpital Ricou, Academic Hospital of Guadeloupe, 97159 Pointe-à-Pitre, Guadeloupe; 9 Laboratoire d’Excellence du Globule Rouge (Labex GR-Ex), PRES Sorbonne, 75015 Paris, France; 10 CAribbean Network of REsearchers on Sickle Cell Disease and Thalassemia, UMR Inserm 1134, Hôpital Ricou, Academic Hospital of Guadeloupe, 97159 Pointe-à-Pitre, Guadeloupe

**Keywords:** sickle cell disease, newborn screening, Caribbean

## Abstract

The region surrounding the Caribbean Sea is predominantly composed of island nations for its Eastern part and the American continental coast on its Western part. A large proportion of the population, particularly in the Caribbean islands, traces its ancestry to Africa as a consequence of the Atlantic slave trade during the XVI–XVIII centuries. As a result, sickle cell disease has been largely introduced in the region. Some Caribbean countries and/or territories, such as Jamaica and the French territories, initiated newborn screening (NBS) programs for sickle cell disease more than 20 years ago. They have demonstrated the major beneficial impact on mortality and morbidity resulting from early childhood care. However, similar programs have not been implemented in much of the region. This paper presents an update of the existing NBS programs and the prevalence of sickle cell disease in the Caribbean. It demonstrates the impact of the Caribbean Network of Researchers on Sickle Cell Disease and Thalassemia (CAREST) on the extension of these programs. The presented data illustrate the importance of advocacy in convincing policy makers of the feasibility and benefit of NBS for sickle cell disease when coupled to early care.

## 1. Introduction

The Caribbean is defined as the geographical region including the Caribbean Sea, more than 700 islands, and the surrounding coasts. The region is located southeast of the Gulf of Mexico and the North American mainland, East of Central America, and North of South America. A wider definition includes Belize, the Caribbean region of Colombia, the Yucatán Peninsula, and the Guyanas (Guyana, Suriname, French Guiana, the Guyana region in Venezuela, and the state of Amapà in Brazil); these areas have political and cultural ties to the region. The Caribbean islands are organized into 13 sovereign states and 17 overseas territories/departments and dependencies, with 43 million inhabitants and at least five official spoken languages.

The Caribbean countries and territories share some common historical features. Less than two centuries after the arrival of Christopher Columbus in the New World, these territories were all under the rules of the European colonial powers (France, United Kingdom, Spain, Portugal, The Netherlands, and Denmark). The introduction of new crops which needed intensive work, such as tobacco, cotton, and sugarcane, led to the development of the transatlantic slave trade. More than 12 million Africans were deported to the New World [1]. One of the consequences of this massive forced migration was the introduction of sickle cell disease (SCD) into the New World. The migration of Indians as indentured laborers when slavery was abolished led to the introduction of the Arabo-Indian haplotype of the β^S^ gene and thalassemia alleles.

The Caribbean occupies a unique position in the history of SCD in the modern era. Indeed, the first case ever described in the Western medical literature by Herricks in 1910 was that of a young fellow from Grenada studying dentistry in Chicago (USA) [2]. Additionally, the benefits of newborn screening (NBS) for SCD as a public health tool, including evidence of both the feasibility of the test and its major impact on mortality and morbidity, were first demonstrated in Jamaica [3]. Available data also suggest that the prevalence of SCD in the Caribbean Region is second only to Sub-Saharan Africa [4].

However, prior to 2006, accurate SCD prevalence and epidemiological data were available for only a limited number of these countries and territories. These data were provided from NBS programs implemented in Jamaica [5] and in the French territories [6], as well as from a prenatal diagnosis program in Cuba [7,8]. Collaboration between medical and scientific Caribbean teams began to increase in 2006, leading to the founding in 2011 of the Caribbean Network of Researchers on Sickle Cell Disease and Thalassemia (CAREST) as a not-for-profit organization [9]. Promotion of NBS for the hemoglobinopathies and assistance for the establishment of sickle cell centers were the primary goals of CAREST. CAREST has worked with all regional stakeholders with common goals, including the SickKids Caribbean Initiative. It has included outreach initiatives to clinicians and policy makers to share regional data regarding the status of SCD NBS and the implications for their own countries. As a result, we present an SCD NBS program initiated in Tobago since 2008 and pilot NBS programs conducted in Grenada and Saint Lucia in 2014–2015 and 2015–2017, respectively.

Beyond the presentation and discussion on the experience of CAREST in establishing NBS programs, we also present an update on the prevalence of SCD in the Caribbean.

## 2. Materials and Methods

### 2.1. Neonatal Screening for Sickle Cell Disease

Blood samples obtained neonatally are collected on Guthrie cards. As shown in Table 1, cord blood samples are collected in Martinique, Jamaica, and St Lucia, whereas in Guadeloupe, Tobago, Grenada, and French Guiana, heel prick samples are obtained. Heel prick samples were also collected for the Saint Lucia’s pilot NBS project funded by the SickKids Caribbean Initiative (2015–2017) and these samples were sent and processed in Jamaica. This project sought to test the feasibility of replacing cord blood sampling and hemoglobin electrophoresis which had been in place since 1992, by heel prick sampling and high-performance liquid chromatography (HPLC); during the pilot, both systems ran concomitantly. Blood samples from Tobago and Grenada are sent by post-mail and analyzed in Guadeloupe (Table 1).

As indicated in Table 1, the samples are primarily tested in reference laboratories based in Guadeloupe (University Hospital of Guadeloupe), Martinique (University Hospital of Martinique), and Jamaica (Sickle Cell Unit, Caribbean Institute for Health Research, Kingston and Southern Regional Health Authority, Manchester). French Guiana specimens are sent to a reference laboratory based in mainland France (regional center for metabolic disease screening, Lille). Saint Lucian cord blood samples, except for those obtained during the pilot study, are tested locally.

NBS itself now primarily uses three laboratory-based methodologies for detecting Hb variants: isoelectric focusing (IEF), capillary electrophoresis (CE), and high-performance liquid chromatography (HPLC), one is used as the first-line screening method and a second as a confirmatory test. In Saint Lucia, however, hemoglobin cellulose acetate and citrate agar electrophoresis are used locally for cord blood samples. In Guadeloupe, DNA analysis is also secondarily performed as a confirmatory diagnosis of a new sample (peripheral blood on EDTA as anticoagulant) in the following cases: FS phenotype when the two parents cannot be tested in order to distinguish SS, S/beta-thalassemia, or S/HPFH; and the FSX or FCX phenotype in order to formally identify the abnormal hemoglobin (HbX), as well as for ambiguous primary screening results [11,12].

### 2.2. Prenatal Screening for SCD

In Cuba, the screening for SCD is a prenatal diagnosis based on fetal DNA analysis. This procedure has been established since 1982 for mothers at risk and the screening program is performed on pregnant women at the provincial centers of genetics located all over the country [7,8].

### 2.3. Initiation of Early Childhood Care for SCD

Children confirmed to have a diagnosis of SCD are enrolled in the different sickle cell centers or clinical structures as soon as possible to initiate clinical management and to provide information to the parents. To shorten delays and promote early medical management of the newly identified SCD children from Tobago, the results are sent by e-mail from the laboratory in Guadeloupe to the appropriate health care provider using secure file transfer systems. Similar communication procedures were also used during the pilot projects in Grenada and Saint Lucia.

### 2.4. Data Analysis

Allele frequencies were estimated by gene-counting and the prevalence of SCD was calculated from the newborn screening results.

## 3. Results

Table 2 summarizes the main results of the hemoglobinopathy NBS programs performed in the French territories [6], Jamaica [5], Grenada [13], Tobago [9], and Saint Lucia [10].

The current coverage of the NBS programs is as follows: Guadeloupe (>98%), Martinique (>99%), Jamaica (>98%), and Tobago (96%). The coverage of the two pilot NBSs in Grenada and Saint Lucia were 79% and 45% respectively. In Cuba, since the beginning of the program until December 2016, 7659 couples at risk have been identified.

The highest frequency of sickle cell trait carriers is observed in Jamaica (9.74–9.94%), Grenada (9.56%), and Tobago (9.32%). In Jamaica, less samples presenting abnormal hemoglobin other than HbS or HbC have been observed during the 2016–2017 period (0.16%) than during the previous screening period (0.64%), probably due to an optimization of the Hb variant detection.

The low screening coverage rate (45%) in Saint-Lucia during the period 2015–2017 could explain the differences in the frequency of phenotypes observed compared to the 1992–2010 period.

Table 3 summarizes the currently available data on β^S^ and β^C^ allele frequencies, carrier prevalence, and SCD prevalence in the Caribbean area.

Several abnormal genotypes, some of which include hemoglobin variants leading to sickle cell disease when associated with the β^S^ allele, have also been identified during the course of these NBS programs. Indeed, the second most frequent sickle cell genotype encountered in these populations was the genotype SC, with some differences in the β^S^/β^C^ ratio detected, and the highest was observed in Saint-Lucia and the lowest in Tobago, as indicated in Table 3. The others correspond to S/β-thalassemia compound heterozygosity (including S/E and S/Lepore) and also S/D^Punjab^ [3,6].

Once infants are screened, confirmation and referral for care are ensued. In French speaking territories and in Jamaica, care is provided according to the guidelines of the French Health Authority [14] and the Sickle Cell Unit [15], respectively. The Sickle Cell Unit Clinical Care Guidelines are currently in use in several other Anglophone countries, including Trinidad and Tobago, Saint-Lucia, the Bahamas, and Barbados. The focus is on pneumococcal prevention and general health maintenance (parents’ education, counselling) prior to the onset of complications. In Martinique, Guadeloupe, French Guiana, and Jamaica, most of the SCD children identified by the NBS program are followed by Sickle cell centers before the age of three months [5,6]. In Saint-Lucia and Tobago, babies identified are followed up in pediatric outpatient clinics. In Cuba, guidelines for management and treatment have also been developed [8].

## 4. Discussion

Given the SCD prevalence and the demonstration of the benefit of the NBS program, one might have expected that SCD NBS would be entrenched across the Region. This is clearly not the case and our data suggest several factors which may be at play, with the availability of resources being a major issue [5,6,9].

Caribbean territories which are part of larger states which mandate SCD NBS as part of a larger universal NBS program, have long and well-established programs. These may be the best funded programs in the Region. In the three French territories, Guadeloupe, Martinique, and French Guiana, the cost of the test is borne by the French government. In territories of the United States of America, screening started in Puerto Rico in 1977 and the US Virgin Islands in 1987 [16]. The test mandated by law covers 99% of births, but the hospitals include a charge to the patient for the NBS panel (http://bft.stage.bbox.ly/newborn-screening/states/puerto-rico). Overseas British territories have separate health systems and SCD NBS is not uniformly offered.

Among the independent nations, Cuba has an integrated public health program and SCD prenatal testing for couples at risk and carrier women was mandated by law in 1983 [7,8]. This program seeks to actively prevent the births of children with SCD, and is perhaps the most active in promoting the termination of pregnancies; since termination of pregnancy was requested by 76.5% of at-risk couples.

In the other independent Caribbean nations, screening varies based on factors such as historical context, current champions, and public health commitment. Jamaica has a unique historical context as the site of the Jamaica Sickle Cell Cohort Study. Nevertheless, after the completion of recruitment for the cohort study in 1981, SCD NBS ceased until 1995, when, through the advocacy of the Sickle Cell Support Club of Jamaica (now the Sickle Cell Support Foundation), it was restarted. For a decade, it was limited to three hospitals in the South East Regional Health Authority, providing screening for approximately 43% of all national births. The coverage gradually increased from 2008 to 2015 when essentially universal coverage was achieved. While testing was mandated in the National Strategic Plan for the Prevention and Control of Non-communicable Disease in Jamaica 2012–2018, there is no legislative mandate and the integration of the program into the fabric of the public health system remains incomplete. Thus, the sustainability of the program depends on the support of incumbent policy makers.

The Saint Lucia Sickle Cell Association (SSCA), a local non-governmental organization, has been strong for many years. It was influential in the introduction of universal SCD NBS in 1992 and its integration into the Ministry of Health’s Community Child Health Service (CCHS) [10]. The program uses hemoglobin electrophoresis to test cord blood samples. A pilot program funded by the SickKids Caribbean Initiative using HPLC testing of heel prick samples had a disappointing uptake and the initial approach continues. The pilot in Tobago has also been successful; it has been continuous for a decade. It is funded by the Regional Health Authority of Tobago, which has made the diagnosis and treatment of SCD a priority. Screening of the immediate family of babies identified with the trait or the disease is done by electrophoresis in the hospital laboratory, thus a greater percentage of the population now know their genotype and there is a greater awareness through education. The initiation of SCD NBS at a major obstetric hospital in Trinidad in 2018 is further evidence of the acceptance of this program.

Pilot screening projects in Grenada [13], St Vincent and the Grenadines [17], and Haiti [18], funded by CAREST, the Medical University of South Carolina (Charleston, SC, USA), and University Hospitals Medical Center (Cleveland, OH, USA), respectively, were not sustained once project funding ended. Local policy makers were not able to identify funding and human resources for continued screening. A pilot posited in Barbados was not undertaken [19]. Instead, screening of pregnant women and testing postnatally of at risk children was the approach chosen.

Currently, champions in Antigua and Guyana are pressing to start pilots and perhaps sustainable programs. These outcomes again indicate the importance of advocacy in convincing policy makers of the feasibility and benefit of SCD NBS. In both Jamaica and Saint Lucia, advocacy groups have been critical to convincing public health officials to initiate and maintain screening. CAREST has a role in supporting their advocacy efforts to secure governmental support and sustainable funding, even as screening begins. In this framework, CAREST has recently obtained funding from the European Regional Development Fund. This funding dedicated to the development of cooperation between the French Departments of the Americas and the other countries/territories of the Caribbean Basin will allow the screening of Grenada to be re-launched, initiate a pilot study for Antigua, and evaluate strategies to ensure the sustainability of this NBS.

The Saint-Lucia and Tobago experience clearly demonstrate that the model of using a few regional laboratories to increase efficiencies of scale, decreasing per cost tests, can be used. Actually, given the high cost of equipment and requisite disposables, and the relatively small populations in the Region, the use of two regional laboratories (Jamaica and Guadeloupe) proved to be a cost-effective approach, once reasonably costed transportation of samples and secure data flows are available. However, it is worthwhile to notice that a significant proportion of the Caribbean populations do not have access to screening program, such as Haiti, which accounts for more than 90% the Francophone inhabitants of the Caribbean, and the Dominican Republic, with approximately 40% of Spanish-speakers. Up to now, only a little more than 50% of the English and Spanish speaking infants are screened.

The cost of national SCD NBS programmes may decrease significantly if efficient, accurate, and inexpensive point-of-care (POC) devices become available; a number of such POC testing devices have recently been developed [20]. These low-cost devices, which must have high specificity to detect HbS and HbC in the presence of HbF and the capacity to distinguish the trait (HbAS) from samples with SCD, must also be easy to use. Two of them, relying on lateral flow immunoassays, the SickleSCAN [21] and the HemoTypeSC tests [22], could be viable screening tools for the early diagnosis of SCD conducted by health workers with little expertise. Preliminary data using the HemoTypeSC test on a small series of children and adults in Martinique, in comparison with a larger series in Ghana and the USA, showed the good specificity and sensitivity of the test [23]. We plan to conduct larger studies to evaluate the performance and implementation feasibility of these POC testing devices as screening tools in Caribbean territories where the reference “gold-standard” tests, IEF, and HPLC are not available. This approach may also reduce the number of samples to be screened by the reference laboratories and the delay between the blood sampling and the transmission of the result and ultimately reduce the age of inclusion of the newly identified children. Mothers would get immediate feedback if their children’s tests are normal, and be advised of the need to do confirmatory tests in cases consistent with traits or SCD. This promising strategy is expected to promote the extension of screening programs, and lead to the clarification of the prevalence and to a better management of SCD in the Caribbean. Audits of important outcomes, such as time to enrollment in clinic, initiation of splenic palpation, and Pneumococcal prophylaxis, as well as continued ongoing ascertainment of survival in countries and territories with SCD NBS, will help to determine what implementation models are most successful and guide the subsequent initiation of programs in other settings.

Differences of SCD prevalence in the Caribbean islands could be observed, with the highest being detected in Jamaica and Grenada and the lowest in Cuba. Various factors may explain these variations, such as the selective introduction of crop production requiring a greater or lesser need for slaves, the settlement policy of the colonial powers with France and the United Kingdom importing few of their own population compared to Spain, for example, as well as the significance of migrations after the end of slavery (ranging from 1804 in Haiti to 1888 in Brazil). In addition, various β^S^/β^C^ ratios have been detected in the studied populations. Since the distribution of the β^C^ allele is more restricted than that of the β^S^ allele in Africa, this data could be related to differences in the African origins of the deported slaves in these territories. These differences in the β^S^/β^C^ ratio could also result from sampling effects; a relatively small number of newborns were screened in some populations. Few epidemiological data from continental countries of the Western coast of the Caribbean Sea coast have been produced so far. An NBS pilot study was conducted in Costa Rica with a total of 70,943 samples and led to the identification of five SS and one SC children [24]. In addition, several clinical reports or genetic studies indicating the presence of SCD in Panama [25], Colombia [26], and Venezuela [27] have been published, but none of these countries have implemented an NBS program on SCD and no accurate data of the prevalence of the disease are available so far, to the best of our knowledge.

In summary, SCD is a perfect example of a disease which fulfils all requirements for doing NBS. Parents are usually asymptomatic and may not know of their risk. Tests done on an asymptomatic baby can allow them to access interventions that decrease morbidity and preventable mortality. CAREST will continue to advocate and work towards universal SCD NBS across the Caribbean Region, regardless of language, per capita income, or political system.

## Figures and Tables

**Table 1:** Neonatal screening process.

Site	Sample	Screening Center	First Test	Confirmation Test
Guadeloupe Tobago Grenada	Heel prick/Guthrie cards	Guadeloupe	HPLC	IEF
Martinique	Cord blood/Guthrie cards	Martinique	IEF	HPLC
French Guiana	Heel prick/Guthrie cards	France (Lille)	CE	HPLC
Jamaica 1995–2015	Cord blood/Guthrie cards	Jamaica	Citrate agar	Cellulose acetate
Jamaica 2015–2018			HPLC	IEF
Saint Lucia 1992–2018 ^†^	Cord blood/Guthrie cards	Saint-Lucia	Citrate agar	Cellulose acetate
Saint Lucia 2015–2017 ^††^	Heel prick/Guthrie cards	Jamaica	HPLC	IEF

^†^ From Alexander et al. [10]; ^††^ From pilot (results not previously published). HPLC: high-performance liquid chromatography; IEF: isoelectric focusing; CE: capillary electrophoresis.

**Table 2:** Results from the newborn screening programs in the Caribbean.

Site	Period	Number of Samples Screened	FAS	FAC	FS	FSC	FC	Other
Jamaica	1995–2006 *	150,803	14,688	5420	557	332	115	972
			9.74%	3.59%	0.37%	0.22%	0.08	0.64%
			9.59–9.89%	3.50–3.69%	0.34–0.40%	0.20–0.25%	0.06–0.09%	0.61–0.69%
	2016–2017	40,444	4020	1481	165	95	35	63
			9.94%	3.66%	0.41%	0.23%	0.09%	0.16%
			9.65–10.24	3.48–3.85	0.35–0.47	0.19–0.29	0.06–0.12	0.12–0.20
Guadeloupe	1984–2010	178,428	14,126	4375	310	231	39	248
			7.92%	2.45%	0.17%	0.13%	0.02	0.14
			7.79–8.04%	2.38–2.52%	0.16–0.19%	0.11–0.15%	0.02–0.03%	0.12–0.16%
Martinique	2009–2015 **	30,171	2134	910	44	29	11	88
			7.07%	3.02%	0.15%	0.10%	0.04%	0.29%
			6.79–7.37%	2.83–3.22%	0.11–0.20%	0.07–0.14%	0.02–0.07%	0.29–0.36%
French Guiana	1992–2013	115,200	8824	2797	293	186	NA	NA
			7.66%	2.43%	0.25%	0.16%		
			7.51–7.81%	2.34–2.52%	0.23–0.29%	0.14–0.19%		
Grenada	2014–2015	1914	183	63	10	2	1	2
			(9.56%)	3.29%	0.52%	0.10%	0.05%	0.10%
			8.32–10.96%	2.58–4.19%	0.28–0.96%	0.03–0.38%	0.01–0.3%	0.03–0.38%
Tobago	2008–2017	7389	689	285	28	14	5	21
			9.32%	3.86%	0.38%	0.19%	0.07%	0.28%
			8.68–10.01%	3.44–4.32%	0.26–0.55%	0.11–0.32%	0.03–0.16%	0.19–0.43%
St Lucia	1992–2010 ^†^	36,253	3146	NA	180	59	NA	NA
			8.68%		0.50%	0.16%		
			8.39–8.97%		0.43–0.57%	0.13–0.21%		
	2015–2017 ^††^	2023	238	42	3	5	2	NA
			11.76%	2.08%	0.15%	0.25%	0.10%	
			10.43–13.24%	1.54–2.79%	0.05–0.44%	0.11–0.58%	0.04–3.6%	

Number of samples screened with the FAS, FAC, FS, and FSC phenotypes are indicated (first line), as well as the prevalence (%) and (95% confidence interval). Other: samples presenting with an abnormal hemoglobin other than HbS or HbC; NA: Not available; * samples collected under the South–East Regional Jamaican Health Authorities only and tested at the Sickle Cell Unit, Caribbean Institute for Health Research (from King et al. [5]); ** universal screening was initiated in Martinique in 1986 in two different laboratories and in 2009, it was centralized in one single center; reliable data are only available from 2009 onwards; ^†^ from Alexander et al. [10]; ^††^ From pilot (results not previously published).

**Table 3:** SCD birth prevalence in the Caribbean countries and territories.

(A) Neonatal Screening					
Country/Territory	Screening Method	Carrier Prevalence (Hb S and Hb C Trait)	Gene Frequencies	β^S^/β^C^ Ratio	SCD Prevalence
Jamaica	Specific locations (1995–2006) [6]	15%	β^S^: 0.055–β^C^: 0.019	2.89	0.53%–1/188
	Univ screen (2016–2017)	13.6%	β^S^: 0.055–β^C^: 0.020	2.75	0.65%–1/153
Guadeloupe	Univ screen	10.5%	β^S^: 0.042–β^C^: 0.013	3.23	0.33%–1/304
Martinique	Univ screen	10%	β^S^: 0.040–β^C^: 0.012	3.33	0.31%–1/322
French Guiana	Univ screen	10%	β^S^: 0.039–β^C^: 0.012	3.25	0.42%–1/235
Tobago	Univ screen	13.2%	β^S^: 0.051–β^C^: 0.021	2.43	0.57%–1/176
Grenada [7]	Univ screen	12.85%	β^S^: 0.054–β^C^: 0.018	3.00	0.63%–1/160
Saint Lucia	Univ screen	13.8%	β^S^: 0.062–β^C^: 0.013	4.77	0.39%–1/253
Haiti [9]	Pilot screen	13.46%	β^S^: 0.059–β^C^: 0.013	4.54	0.58%–1/173
Saint Vincent & Grenadines [10]	Pilot screen	15.27%	β^S^: 0.065–β^C^: 0.016	4.06	0.26%–1/382
**(B) Prenatal Diagnosis**					
**Country/Territory**	**Screening Method**	**Carrier Prevalence (Hb S and Hb C Trait)**	**Gene Frequencies**	**β^S^/β^C^ Ratio**	**SCD Prevalence**
Cuba	Prenatal diagnosis	3.1%	β^S^: 0.011–β^C^: 0.0036 ^a^	3.06	
			β^S^: 0.053–β^C^: 0.006 ^b^	8.83	0.02%–1/5000

Univ screen: universal screening; ^a^: Figures for the Western side of Cuba (not including for Havana); ^b^: figures for the Southeastern side of Cuba.
